# What medical students and residents learned from reflection through patients’ perspectives: a qualitative study

**DOI:** 10.5116/ijme.6741.f16c

**Published:** 2024-12-05

**Authors:** Kazuki Tokumasu, Puthiery Va, Haruo Obara, Lisa Rucker

**Affiliations:** 1 Department of General Internal Medicine, Okinawa Chubu Hospital, Uruma, Japan; 2 Department of Medicine, Albert Einstein College of Medicine/Jacobi Medical Center, Bronx, NY, USA

**Keywords:** Awareness, cognitive process, patient’s perspective, per-son-centered care, qualitative study, reflection

## Abstract

**Objectives:**

The purposes of this study were to identify reflective processes from patients’ points of view for difficult patient-doctor interactions and learn how the processes made medical students and residents aware of their own medical practice. These processes were compared in two countries (US and Japan).

**Methods:**

The study was a descriptive qualitative study utilizing semi-structured interviews. Participants were from US and Japan. We analyzed the data using reflective thematic analysis of the implementation of reflections (from a patient’s point of view) into medical clinical education and training from a constructivism paradigm.

**Results:**

We included twenty participants each from US and Japan by convenience sampling.  The participants were medical students during clinical clerkship and post graduate year-1, 2, and 3 medical residents. Medical students and residents realized four cognitions (different expectations of patients and doctors, language communication barrier, time restriction and healthcare system challenges) from reflections from patients’ perspectives. Subsequently, medical students and residents identified three types of awareness (appropriate communication, empathy, and patient-doctor relationship of trust).  During these reflections from patients’ perspectives, the medical students and residents had a willingness to change future behavior.

**Conclusions:**

This study revealed one aspect of medical students’ and residents’ cognitions and awareness of clinical experiences through reflection from patients’ perspectives.  This cognitive process aligns the ideas of medical students and residents more closely with the patients’ perceptions and influences their willingness to change their behavior. This process enables comprehensive realization in person-centered care.

## Introduction

Reflection is a metacognitive process that creates a greater understanding of both the self and the situation so that future actions can be informed by this understanding.^1^  Reflection can also contribute to learning that consists of emotional and social dimensions as well as cognitive dimensions.^2^  In clinical practice, the cognitive aspects are most easily measured through examinations or performance assessments, while the emotional and social aspects may be less easily captured.^3^^,^^4^ Frameworks of reflection, such as reflective processes as a model, could support the development of both dimensions.^4^  This model requires three steps: awareness of uncomfortable feelings and thoughts, critical analysis of feelings and knowledge, and new perspective.^4^

As reflective practitioners, doctors use reflection in their practice because reflection can identify deficits in performance and give cues for specific ways in which to do better.^5^^, ^^6^ When conducting reflective practice, it is important to know what kind of reflection one is conducting.  For instance, educational reflection often is performed in the clinical setting^7^^, ^^8^ according to Kolb’s Learning Cycle, based on the social cognitive theory.^9^  However, these reflections are performed from a practitioner’s perspectives, especially medical errors or mistakes.^10^^, ^^11^  On the other hand, reflective practice is also important in practicing patient-centered medicine, which is considered to be one of the higher quality medical practices.^12^  In practicing patient-centered medicine, it is necessary to interpret medical activities from the patient's perspective.  Reflection on empathy in medical education could provide learners with considering patients’ history, thoughts and emotions deeper,^13^^,^^14^ emphasizing the relational domain in person-centered care.^15^ 

There is the psychological concept of Self-Reflection and Perspective Taking.^16^   This concept emphasizes self-reflection by the adoption of another person’s perspective. This is a process of self-reflection in which understanding of one’s own emotions, cognitions, feelings and behaviors, led to greater interpretation of other person’s perspective.^16^  This framework was based on previous literature focused on self-reflection including self-awareness^17^^,^^18^ and self-consciousness,^19^^,^^20^ and empathy.^21^^,^^22^  There have been few studies in which the processes of reflective practice from another person’s perspective were comprehensively investigated.

The purpose of this study was to provide answers for the following research questions: 1. Has a difficult patient-doctor interaction, viewed from the patient's perspective, made medical students and residents aware of their own medical practice? 2. What kind of learning points can be learned by reflecting from the patient's perspective?

## Methods

### Study design

We conducted a qualitative analysis of the implementation of a narrative reflective practice from a patient’s point of view into medical education and training.  With informed consent, participants (medical students and residents rotating in the outpatient department) were given a brief explanation about the purpose of the study and then they documented participants’ characteristics (age range, medical school and training, sex).  Then the participants were asked to reflect – from the patient’s point of view- on an interaction with an ambulatory patient that did not go well from the patient’s point of view.  We had interviews using the interview guide (Appendix).

### Participant recruitment and sampling

Participants were gathered by convenience sampling relying on population members who were conveniently available to participate.  Twenty persons (medical students rotating in internal medicine and medical residents) participated in each country at Hospital A (US) and Hospital B (Japan), both of which are teaching hospitals and provide acute care.  Interviews with the participants were conducted from March 2016 to August 2016.

All participants were informed by documents, and all interview memos will be destroyed upon completion of this study.  The Ethics Committee of Jacobi Medical Center/ Albert Einstein College of Medicine (Approval number, 2015-5843) and Okinawa Chubu Hospital (Approval number, H27-62) reviewed and approved this study.

### Setting

This study was conducted at a teaching hospital in the US (Hospital A) and a teaching hospital in Japan (Hospital B).

The US and Japan are appropriate countries for this research because they both have advanced structures and systems of health care and well-designed medical education.^23^^-^^27^

Hospital A is a 450-bed acute care public hospital in Bronx, New York.  The department of medicine has approximately 30 new residents and 70 medical students annually in general medicine services. Approximately 90 attending physicians teach and supervise the learners and also see their own patients.

Hospital B is a public 550-bed tertiary care teaching hospital with a large and active emergency department in central Okinawa.  The hospital accepts about 30 new residents and 150 medical students for clinical training every year.  There are also about 110-120 attending physicians who provide clinical education for residents and medical students.

### Consent and data collection

We obtained informed consent with a consent sheet for participation in this study.  Data were obtained by responses written by the participants or by conducting qualitative interviews.  Each interview lasted approximately 20 to 30 minutes.  All interviews were recorded in interview memos, and the memos were stored in a locked cabinet.  Interviews in Japanese were translated into English and co-researchers reviewed and accepted them.

### Data analysis

We conducted a qualitative analysis of 40 compiled narratives of reflective practice from internal medicine learners in a single center in the US and a single center in Japan. The narratives of reflective practices were analyzed using reflective thematic analysis.^28^^-^^31^

We chose this approach because of its straightforward analytical process.  Two key features of this approach are its efficiency and validity with respect to qualitative data analysis and theoretical coding as well as its theorizing based on relatively small data sets within the paradigm of social constructivism.^32^ The narratives written in Japanese were translated into English by the principal researcher (KT), and the accuracy of the descriptions was checked by a co-researcher (HO).  The other English original narratives were rechecked by co-researchers (VP and LR); KT and VP independently analyzed the qualitative data, and co-researchers (HO and LR) as independent auditors read all the records and reviewed each step of the analysis.  In addition, all of the researchers (KT, VP, HO, and LR) checked all of the codes and concepts.  All researchers held discussions leading to consensus multiple times.  Through the discussions, new codes emerged or codes were modified from different perspectives (US and Japanese backgrounds).  All authors acknowledged the new codes and concepts resulting from this iterative process at the end of the analysis. Finally, the conceptual structure of each of the medical students' and residents' self-reported reflections was identified.

## Results

The characteristics of the participants in Hospital A and Hospital B are shown in Table 1.  The number of female participants in the US was larger than that in Japan and the doctors in the US were older than those in Japan.  Common themes taken from residents’ and medical students’ cognitions from patients’ perspectives are identified in Table 2.  After the reflection, medical students and residents realized three themes shown in Table 3.  After the reflections from patients’ perspectives, medical residents and students reported a willingness to change their behavior.

**Table 1:** Characteristics of the participants

Characteristics		Hospital A, US	Hospital B, Japan
		number (%) (n = 20)	number (%) (n = 20)
Gender			
	Male	12 (60)	16 (80)
	Female	8 (40)	4 (20)
Age group			
	21-25	1 (5)	5 (25)
	26-30	13 (65)	10 (50)
	31-35	6 (30)	5 (25)
Training year			
	Medical student (Clinical training year)	5 (25)	4 (20)
	PGY-1	2 (10)	3 (15)
	PGY-2,3	13 (65)	13 (65)

PGY: Post graduate year

During the reflective process, four themes (different expectations of patients and doctors, language communication barrier, time restriction and healthcare system challenges) taken from residents’ and medical students’ cognitions from patients’ perspectives were identified (Table 2).

### Different expectations of patients and doctors

It is necessary for patients to have appropriate and sufficient explanations about examinations and to understand the explanations.  Medical residents tend to focus on medical problems rather than psychosocial problems.  A Japanese resident realized the importance of cost-consciousness when patients pointed out the high costs of tests.

“… I added the examination. I explained to the patient that the extra examination costs more, but I did not know the exact cost.  On the next outpatient day, the patient asked to me “Why was the exam so expensive!?”....” [No. 7, Hospital Japan, Male, age 26-30, Resident]

An American resident realized the importance of accepting that there were different expectations of patients and doctors.  The doctor’s explanations of the drug’s side effects from biomedical aspects made the patient upset. The interactions helped the residents realize the patient’s feelings, thoughts and relationship with the primary care doctor.

“I saw a patient in the clinic for the first time.  She had another resident for a very long time whom she trusted.  She asked me to prescribe tramadol for chronic knee pain…I explained to her the risk of long-standing opiates and sedatives.  She became very upset when I refused to prescribe them.  When I explained the side effects to her, she said that she only takes them as needed and not daily.  She told me her primary care physician knows her well and trusts her enough that she will only use as needed.  She left very upset after my visit.” [No. 20, Hospital US, Female, age 21-25, Resident]

Feedback from a patient can make the resident realize the patient’s expectations.  Medical practice that was not in line with the patient's expectations led to the interruption of continuing medical care.

“…The emergency doctor assessed him and he had asymptomatic seizure.  But I found many other medical problems including systolic ejection murmur, pitting edema in the lower extremities, hypertension and urinary problems.  My pace of examinations slowed down for further evaluations, and my other patients had to wait for a long time.  When I asked him to take more examinations for further evaluations, he got annoyed.  He was a very elderly person, and I wonder now if it was really what he wanted to do.  Finally, he said to me, "Please introduce me to other hospitals.". [No. 8, Hospital Japan, Male, age 26-30, Resident]

### Language communication barrier

Since there are many different races in the United States, different languages sometimes made communication difficult.

“A 79-year-old woman visiting from Nigeria (non-English speaking) …The difficulty during the clinic encounter was first that family had initially refused a phone interpreter.  When a phone interpreter became available, the interpreter had difficulty understanding patient.  The interaction with the patient took about an hour, and I felt there was a language barrier as well as cultural and educational barriers and possibly some distrust by one of the family members.” [No. 18, Hospital US, Female, age 26-30, Resident]

### Time restriction

Difficulties arose from the physician’s examination time and the patient’s waiting time.

A Japanese medical student worried that a patient would be angry because the patient waited a long time and the medical student felt apologetic because it took a long time to get a physical examination.

“…I was finding a solution to satisfy the patient within a restrictive time.  Before I started the examination, the patient had been waiting for a long time.  So, she was very angry…” [No. 3, Hospital Japan, Female, age 26-30, Medical Student]

**Table 2:** Types of residents’ and medical students’ cognitions from patients’ perspectives

Themes	Different expectations of patients and doctors	Realize differences in expectations of patients and doctors
	Language communication barrier	Different languages of the mother country, appropriate and sufficient explanations
	Time restriction	Long waiting time for patients Psychological pressure that medical students and residents face to work faster and faster
	Healthcare system challenges	Problems of the healthcare system and hospital rules

**Table 3:** Types of awareness from reflection

Themes	Appropriate communication	Emotional control, especially control of anger
	Empathy	Understanding the difficult situation of patients, reflecting on own behavior, and considering whether it is appropriate or not for the patient's condition
	Trust	Interpersonal relationship of trust (patient and physician)

Japanese residents felt mental pressure that they had to work faster and faster.  They realized the examination time was restrictive.

“…I felt pressure to examine patients as soon as possible…” [No. 5, Hospital Japan, Male, age 31-35, Resident]

An American resident realized that waiting a long time led to patient anger and made the doctor-patient interactions difficult.  The patient did not try to communicate with the resident at the first encounter because of anger.  This emotion was caused by waiting for a long time.

“A patient who had been waiting for a long time to see me was upset from the initial encounter.  She wanted to leave as soon as she stepped into the exam room.  She didn't seem to care what I needed to discuss. [No. 8, Hospital US, Male, age 26-30, Resident]

### Healthcare system challenges

An American resident felt that the healthcare system itself was responsible for difficult interactions.  She encountered difficulties in terms of patient participation in the insurance system and the rules for healthcare in the municipality in which the patients live.

“…Finally agrees to have better control of her diabetes mellitus and will promise to start checking her fasting blood sugar level. …However, the patient was uninsured and she wanted a written prescription to pick up the glucometer, strips and needles.  After lengthy discussion of New York City law, mandatory electronic prescription, she finally agreed to come back to give us the information.  Patient was upset saying that this won't help her manage (her diabetes) or get the things she needs to help with her diabetes. [No. 1, Hospital US, Female, age 31-35, Resident]

Residents did not have enough time to give patients adequate explanations.  There are limitations of the hospital function and the residency education system.

A Japanese resident felt that the system in the hospital for blood test was not appropriate.  This triggered the patient’s anger.

“…the patient said to me," You take my blood many times!".  I did not explain the necessity of the blood test.  I recognized the lack of explanations about the blood tests.  It is usual to explain the necessity (of the blood tests) using documents.  The non-efficient system is a responsibility of the hospital.” [No. 11, Hospital Japan, Male, age 26-30, Resident]

Three types of awareness (appropriate communication, empathy, and interpersonal relationship of trust) from reflection are shown in Table 3.

Appropriate communication: Importance of emotional control, especially anger.

In clinical practice, it is important for medical residents to communicate with patients appropriately.  Emotions that arise during communication, especially anger, can make the residents realize the need for emotional control.

When a clinical situation that the medical resident did not expect arises, feelings of anger might develop and communication might become difficult.  A resident got angry because the patient changed his mind.  Thus, the resident felt it was a waste of time to contact the patient. In the interaction with the patient, the resident reflected on how he should control his angry emotions.

“A patient presented to W clinic for a variety of issues, the most pressing was for refills of medicines and referrals for appropriate specialists… While the doctor typed the list, the patient changed his mind about not seeing a doctor…. Then he changed his mind again and said he didn't know what to do.  What would the doctor do? The doctor's anger flashed, so he snapped, you can do whatever you wish! Do you want the referral or not? …The patient rather weakly said, "I'm sorry for wasting your time, please make the referrals."  My anger reflected my stress in taking too much time…I wanted to empower the patient to make his own decision but didn't want him to take any time to decide where he should receive his care …a very important decision.  I should have held my anger and stress in check, pleasantly informed him that he will have the referral no matter what and he could keep or discard the referral at his convenience.  I should have asked also asked for help sooner…” [No. 7, Hospital US, Male, age 31-35, Resident]

### Empathy

Medical residents and medical students understood the difficult situations of patients.  This realization was derived from empathy.  They were aware of the importance of empathy through interactions with patients and reflections from patients’ perspectives.  When an American resident experienced a patient’s reaction that was different than expected, the resident considered the patient’s perceptions and situations.  The patient’s dissatisfaction and distrust made the medical resident’s attitude become empathic.

“An approximately 60-year-old Caucasian male came…He opposed my recommendation suggesting…I feel like he was probably frustrated at the fact that he had to face more medical diagnoses that were thrown at him and that he would have to start taking several different medicines that were suggested by different doctors.  Also, he probably did not trust these "young" doctors seeing him.  I probably should have inquired more from him about what he knew about these diagnoses I was talking about before suggesting any treatment. [No. 15, Hospital US, Female, age 31-35, Resident]

A Japanese medical student reflected on his behavior and was considering whether it was appropriate or not for the patient's condition.  During this process, the medical student demonstrated empathic consideration.

“…I frequently take a medical history with a smile.  Most of the patients get better in the outpatient department setting.  However, some patients get worse.  In this situation, it is not better to communicate with a smile at first contact when the patient seems serious.  I realized that it is very important to imagine the patient’s situation.  This reflection in this session made my ideas clearer by expressing my thoughts.” [No. 4, Hospital Japan, Male, age 21-25, Medical Student]

A medical student touched on the patient's feelings and learned that he had to think carefully about the situation of the patient.

“She was an 85-year-old woman with a chief complaint of insomnia.  She did not sleep at all last night…she was mildly angry. …When I asked her about her appetite, she was angry and said, "I had an appetite loss! I said this many times!"…In this case, I was finding a solution to satisfy the patient during the restrictive time.  The patient waited a long time for my examination.  So, she was very angry.  The patient's point of view is very important when considering the patient's symptoms.…From this reflection session, I realized one of my conflicts as a medical student.” [No. 3, Hospital Japan, Female, age 26-30, Medical Student]

### Trust

A resident recognized the importance of building an interpersonal relationship of trust (patient and physician).  This awareness triggered obstacles by miscommunication.  This miscommunication was caused by a language barrier.

“A 79-year-old woman visiting from Nigeria (non-English speaking)…The difficulty during the clinic encounter was first that the family had initially resisted phone interpreter… The encounter took about an hour, mostly I felt there was a language barrier on top of cultural, educational and possibly some distrust by one of the family members.” [No. 18, Hospital US, Female, age 26-30, Resident]

Doctors have to confirm that the patients understand.  Miscommunication led to a loss of trust, and the resident therefore became aware of the importance of a trusting relationship with the patient.

“…the patient asked me “Why was the exam so expensive!?” I felt it was very important for me to consider the patient’s feelings.  There was a gap between the doctor's perception and the patient's expectation from the medical charges…I realized that the physician’s understanding of the patient’s perspective could foster close relationship with the patient.  Thus, this led to the patient’s trust.” [No. 7, Hospital Japan, Male, age 26-30, Resident]

A Japanese resident realized from a patient’s remarks that the patient did not trust him, and the resident did not have any idea about the patient's perspective.  This was a reflective process to re-recognize the importance of patient-doctor trust.

“One of my patients was avoiding a laboratory check in the morning.  He asked me "Why do you do blood tests every day?".  I could not explain the appropriate reasons.  The relationship between doctors and patients is very important…” [No. 9, Hospital Japan, Female, age 26-30, Intern]

After these reflections from patients’ perspectives, the residents and medical students showed a willingness to change their behavior.

After an intern felt difficulty in communication, the preceptor took the patient’s full complaints. This communication led to a realization by the intern that doctors should make judicious use of interpreter service and not try to ignore minor complaints from patients.  The intern realized that a language barrier caused failure to discover the most important complaints.

“I had a patient who spoke English and another language…I had to use a translator to assess the history but she could not give me a 'complete' history.  I went to my preceptor, and he came back with me to the room and he was fluent in Spanish.  When he interacted with the patient face-to-face in Spanish, she broke down and mentioned that depression was her real problem.  I would probably request to speak to someone (preferably a provider) who is fluent in my language in person (face-to-face and not by using an interpreter).  If there are signs of depression and the patient does not speak English, it would be useful to bring in someone who is fluent in the patient’s language so that they feel more comfortable opening up about their issues.” [No. 6, Hospital US, Male, age 26-30, Intern]

When a resident realized a huge gap between the patient's and doctor's understanding of the rationale for a routine procedure, the resident decided that he would change his behavior in the future and offer an explanation.  Some Japanese patients depend a lot on their doctors regarding treatment policy and they may not understand their disease pathology.

“A patient was admitted with hypokalemia and QT prolongation.  I checked his ECG (electrocardiogram) every day…he asked me ““Why do you check the ECG every day?”” I realized he could not understand the meaning of checking an ECG every day because I did not give him a sufficient explanation about his disease and medical condition.  There was a huge gap between the patient's realization and doctor's cognition of patient's realization...Whenever I encounter a similar patient, I will explain the details of the disease, its condition, and its factors.” [No. 6, Hospital Japan, Male, age 26-30, Resident]

A Japanese resident realized from a patient’s remarks that the patient did not trust him and the resident did not have any idea about the patient's perspective.  This was a reflective process to re-recognize the importance of patient-doctor trust.

“…He asked me "Why do you do a blood test every day?".  I could not explain the appropriate reasons.  The relationship between doctors and patients is very important (in clinical practice)…I always make patients wait a long time. However, I should not forget the patient's point of view.” [No. 9, Hospital Japan, Feale, age 26-30, Intern]

A resident realized the importance of a long-term relationship with the patient and tried to establish such a relationship.

“…Build a long-term relationship with patient is more important than sell her idea during one visit and no need to argue with patient about facts.  Attitude is to serve patient and try to build long term relationship.” [No. 2, Hospital US, Male, age 31-35, Resident]

## Discussion

This study clarified the process of reflection by medical students and residents from a patient’s perspective and how reflections shape future actions.  The reflection examined in this study is also an aspect of describing the cognitive process of medical students and residents.

Superior metacognition has been reported to be associated with better learning outcomes,^33^ and how reflection promotes metacognition is an important element in the learner's learning process.^3^  Furthermore, metacognition as awareness and cognition control also regulates the learner's behavior.^34^^, ^^35^  There is also reflective practice in the field of medicine, but with an emphasis on aspects of medical knowledge and skills such as missed diagnoses and failed procedures.^36^  Reflection on empathy, one of a similar concept similar to reflections from patients’ perspective, focuses on patients’ feelings and thoughts.^13^^,^^14^  This reflection emphasizes the relationship between a physician and the environment that surrounds the physician (patients, colleagues, organizations, etc.).^13^  This concept aligns closely with the relational domain, one of the three domains of person-centered care (epistemic domain, organizational domain, and relational domain).^15^  It is important to note that there is an underlying assumption that empathy is a crucial attribute for doctors, particularly when considering reflection as relationships.^13^^,^^14^  However, this study showed that reflective practice from patients’ perspectives could contribute to the epistemic domain within person-centered care.  Reflections from the patient’s perspective could align the ideas of medical students and residents more closely with the patients’ perceptions.  It might support epistemic reciprocity, which is a key principle that guides clinical negotiations and explores the co-creation of knowledge between patients and doctors in person-centered care.^15^

Another important point of this study was clarification of the process by which key concepts in person-centered care emerged in real-life clinical practice for medical students and residents beginning their clinical careers. This reflection from the patient’s perspective is more clinical and practical, and it is based on the same assumptions as those outlined in Table 3.  These include underlying cognitions shown in Table 2 (differing expectations between patients and doctors, language communication barriers, time constraints, and challenges within the healthcare system) that influenced medical students’ and residents’ awareness.

Medical students and residents are starting out in clinical practice and have unique cognitive processes as both learners and workers in clinical practice.  For example, a study on the internal motivational processes of young doctors showed that there were cognitive processes that proceeded due to external stimulation (a self-handle environment and a near-peer role model) because the young doctors were just starting out in clinical practice.^37^ The process continued to the next cognitive process: gap recognition, awareness of important attitudes as a doctor (autonomy, responsibility, and independence), and internalization.^37^ Thus, reflection, based on one’s own clinical experience may internalize awareness, leading to enhanced clinical expertise in future instances.  In this study, we identified one aspect of the medical students’ and residents’ clinical reflective process from the patient’s perspective, supported by the theoretical background of self-reflection and perspective-taking.

This study also clarified the challenges in medical education. Medical students’ and residents’ reflections from patients' perspectives enabled interpretation of patients’ behaviors and reactions. Difficulty in interpreting the patient's perspective might be attributed to medical education. Medical students tend to focus on biomedical issues.^38^^,^^39^ Therefore, they might emphasize biomedical examinations, diagnoses and treatments.  However, as the results of this study showed, medical students and residents realized that actual clinical care is based on the patient's perceptions and experiences. Patients have a life they have lived up to that point, a background, a culture and a set of values on which their behavior depends.^40^^-^^42^ In other words, not only biomedical aspects but also psychosocial aspects are an important elements of decision-making in healthcare. When clinical experiences are based on the medicine they have studied so far, learners experience the realization of the importance of psycho-sociological aspects. This is because clinical practice deals not only with biomedical aspects but also with the human, or social, being of the patient.  This gives an awareness of the patient not as a biomedical element but as a being that also has psychosocial existence, which can lead to the existing bio-psychological-social model.^43^ This may be an important component of performing person-centered medicine.  Reflection from the patient’s perspective enables comprehensive practice in person-centered care. A novel and unique aspect of this study was the clarification of this reflective process.

Participants in this study were recruited from two culturally distinct countries, the USA and Japan.  Therefore, it is important to consider the different cultural backgrounds of the participants.  Among the various types of residents’ and medical students’ cognitions from patients’ perspectives in this study, a language communication barrier was a unique cognition characteristic in the USA.  This is because the USA is a multi-ethnic country with numerous mother tongues, leading to communication barriers.^44^^, ^^45^  On the other hand, Japan is predominantly mono-racial, with over 97% of the population being Japanese.^46^  This is largely due to the influence of isolation, which lasted for more than 200 years from the 1600s to the 1800s during the Edo period.^47^^, ^^48^

Although the experiences underlying various types of cognition differed depending on the country, there were many similarities in awareness.  This suggests that across countries, ethnicities and cultures, there are many similarities in metacognition by reflection of medical students and residents soon after entering clinical practice.

### Implications for clinical practice

Reflections from patients’ perspectives in the present study have the potential for comprehensive interpretation of the patient.  This would lead to person-centered care.^15^

This study was an exploratory study, and further research is needed to determine what kind of cognition of medical students and residents is promoted by a specific reflection, what kind of behavioral changes are linked to this cognition, and what kind of patient outcomes are linked to this cognition.

### Limitations

In interpreting the results of our study, methodological and cultural limitations must be considered.  First, because this study was an exploratory study, the findings obtained represent only some aspects of the cognitive processes of medical students and residents in their clinical experiences from the patient's perspective.  Second, there was limited research diversity.  Even if there were homologies in the educational programs of medical students and residents, the perspective backgrounds of the patients would be influenced by culture and would differ.  The diversity of the study's findings was due to recruitment of participants from two very different cultures, Western and Eastern cultures.  In order to consider the diversity of further research questions, it will be necessary to recruit participants from several different countries and cultures.  Third, this study revealed the reflections of medical students and trainee doctors from the patient’s perspective.  However, it did not include the patients’ opinions and narratives.  This is why this study is unable to validate whether the interpretations of the patient’s thoughts and feelings, as perceived by the medical students and residents, align with the patients’ perspectives.  Fourth, convenience sampling was used for recruiting medical students and residents, which may have generated a selection bias.  Finally, because two authors conducted the interviews and four authors analyzed the qualitative data, the possibility of confirmation bias could not be eliminated.  To minimize the possibility of this bias, we repeated discussions to achieve a consensus regarding the results.  The principal researcher and co-authors thoroughly checked each step of the analysis from different perspectives and revisited codes and themes.

## Conclusions

This study revealed one aspect of medical students’ and residents’ cognitions and awareness of clinical experiences through reflection from patients’ perspectives.  This cognitive process influences the willingness to change their behavior.  This process will enable comprehensive realization of person-centered care.

### Acknowledgment

We would like to thank all the participants and the medical staff of Okinawa Chubu Hospital and Albert Einstein College of Medicine/Jacobi Medical Center for their contribution to the management of the educational and training environment.

### Conflict of Interest

The authors declare that they have no conflicts of interest.

## Supplementary material

Supplementary file 1 Appendix. Interview guide
