# Supplementary material for: What medical students and residents learned from reflection through patients’ perspectives: a qualitative study
Source: Int J Med Educ. 2024 Dec 5;15:150–8. doi: 10.5116/ijme.6741.f16c (PMC11687375; doi:10.5116/ijme.6741.f16c)
Supplement: Supplementary file 1 — Appendix. Interview guide [file ijme-15-150-S1.pdf]

Appendix

Interview guide

Interview guide
1. Summarize the interaction that did not go well.
2. After reflecting on the interaction, what would you do differently if faced with the same problem in the future?
3. What did you learn from this exercise in reflection?
